# Is Pesticide Use Related to Parkinson Disease? Some Clues to Heterogeneity in Study Results

**DOI:** 10.1289/ehp.1103881

**Published:** 2011-10-21

**Authors:** Marianne van der Mark, Maartje Brouwer, Hans Kromhout, Peter Nijssen, Anke Huss, Roel Vermeulen

**Affiliations:** 1 Institute for Risk Assessment Sciences, Division of Environmental Epidemiology, Utrecht University, the Netherlands; 2 St. Elisabeth Hospital, Tilburg, the Netherlands; 3 Julius Centre for Public Health Sciences and Primary Care, University Medical Centre, Utrecht, the Netherlands

**Keywords:** exposure assessment, fungicides, herbicides, insecticides, meta-analysis, Parkinson disease, pesticides, systematic review

## Abstract

Background: Previous systematic reviews have indicated that pesticide exposure is possibly associated with Parkinson disease (PD). However, considerable heterogeneity has been observed in study results.

Objective: We aimed at providing an update of the literature published on PD and exposure to pesticides by performing a systematic review and meta-analysis. In addition, we investigated whether methodological differences between studies could explain the heterogeneity in study results.

Methods: We identified studies through a systematic literature search. We calculated summary risk ratios (sRRs) for pesticide exposure and subcategories using random effects meta-analyses and investigated sources of heterogeneity by meta-regression and stratified analyses.

Results: Thirty-nine case–control studies, four cohort studies, and three cross-sectional studies were identified. An sRR of 1.62 [95% confidence interval (CI): 1.40, 1.88] for pesticide exposure (ever vs. never) was found. Summary estimates for subclasses of pesticides indicated a positive association with herbicides and insecticides, but not with fungicides. Heterogeneity in individual study results was not related to study design, source of control population, adjustment of results for potential confounders, or geographical area. However, results were suggestive for heterogeneity related to differences in the exposure assessment. Job title–based exposure assignment resulted in a higher sRR (2.5; 95% CI: 1.5, 4.1) than did assignment based on self-reported exposure (e.g., for self-reported ever/never exposure, sRR = 1.5; 95% CI: 1.3, 1.8).

Conclusions: This review affirms the evidence that exposure to herbicides and insecticides increase the risk of PD. Future studies should focus on more objective and improved methods of pesticide exposure assessment.

Parkinson disease (PD) is an idiopathic degenerative disorder of the central nervous system that impairs motor skills, cognitive processes, and other functions. The etiology of PD is largely unknown, although some genetic factors have been identified (Bekris et al. 2010; Shulman et al. 2011). Based on published epidemiological and toxicological studies, pesticides may be involved in the etiology of PD (Brown et al. 2006). However, epidemiological evidence is far from conclusive, as considerable heterogeneity has been observed in study results (Brown et al. 2006; Li et al. 2005; Priyadarshi et al. 2000). Possible methodological causes of heterogeneity in study results have been suggested and include differences in study design, control selection, diagnosis of patients, and statistical analysis (Brown et al. 2006). Differences in exposure assessment methods could contribute to heterogeneity as well. Most previous studies relied almost exclusively on self-reported exposures, a process that is prone to recall bias, especially in case–control studies, and could lead to false-positive associations. Alternatively, one could speculate that PD patients might underreport pesticide exposure because of cognitive deficits, leading to false-negative associations. Furthermore, differences in the definition of exposure to pesticides (occupational vs. nonoccupational use, ever/never vs. regular use) could also result in heterogeneous study results. Lastly, the regions where the studies have been conducted could be of importance as regulation, types, and use of pesticides may differ from region to region.

Several recent studies have been published on pesticide exposure and PD risk, including some prospective (cohort) studies. In the present analysis, we aimed at providing an update of the literature published since the last systematic review on PD (Brown et al. 2006) and exposure to pesticides and pesticide subcategories by performing a systematic review and meta-analysis. We specifically set out to address the question of whether the previously described heterogeneity in study findings could be explained by differences in study design and exposure assessment methods.

## Methods

*Data source.* We searched the databases Embase (http://www.Embase.com/), starting with 1974, and Medline (http://www.ncbi.nlm.nih.gov/pubmed/), starting with 1950, through November 2010 using the search term “Parkinson” in combination with “pesticide*,” “insecticide*,” “fungicide*,” “herbicide*,” “rodenticide*,” “organochlorine*,” “organophosphate*,” “carbamate*,” “glyphosate*,” “paraquat,” “maneb,” “lindane,” “dieldrin,” “rotenone,” “DDT,” or “environmental factors.” The search was limited to publications in English, French, German, or Dutch; to human studies; and to original publications. We also searched the reference lists of the retrieved publications.

*Study selection.* We included studies that specifically investigated PD or parkinsonism. We included cohort studies, case–control studies, and cross-sectional studies. No reviews, case reports, or conference abstracts were included. We excluded studies that summarized results of pesticide exposure only within a broad category of “chemical exposure.” Exposure to pesticides was defined as use of pesticides by the subject, thus excluding environmental studies.

*Data extraction.* Two reviewers (M.M., M.B.) independently extracted reported risk estimates [i.e., odds ratios (ORs), risk ratios (RRs), or prevalence ratios], study designs, exposure assessment methods, and types of source population for the controls. We also evaluated subcategories of pesticides and extracted data about exposure–response relations and individual pesticides. Two other researchers (A.H., R.V.) acted as referees in cases of any differences. If authors reported adjustment for potential confounders, we preferred adjusted risk estimates over crude risk estimates. In cases where no risk estimate or 95% confidence interval (CI) was reported, we calculated crude risk estimates and 95% CIs with the reported numbers. Where risk estimates were reported separately for men and women, we pooled the risk estimates with a within-study meta-analysis (Vlaanderen et al. 2011). Of studies with more than one control group, the results of population controls were preferred above the results of hospital controls because population controls are generally considered to be a more representative comparison group than hospital controls.

*Statistical analysis.* Because of the observed heterogeneity in study results, we conducted a DerSimonian and Laird (1986) random effects meta-analysis to pool the results of the separate studies for risk for pesticide exposure and the subgroups of herbicides, insecticides, and fungicides. We also stratified by whether or not nonoccupational exposure (e.g., gardening) was included in the exposed group. This was because of potential differences between occupational and nonoccupational exposures in intensity and frequency of exposures. In one publication, results both for occupational and for occupational and/or nonoccupational exposure were reported (Frigerio et al. 2006). We chose to include risk estimates of the more inclusive exposure definition, although final results did not differ when we included the risk estimates based on only occupational exposure (data not shown).

Subsequently, we explored whether heterogeneity in observed risk estimates could be explained by study and exposure assessment characteristics. We did so by stratification and used meta-regression to explore statistical significance of these characteristics. Given the limited number of studies, we only explored one characteristic at a time. Characteristics explored were the type of exposure assessment (self-reported ever/never pesticide exposure, self-reported regular pesticide exposure, or exposure assessment based on reported job titles by expert judgment and/or applying a job-exposure matrix), source of control population [hospital, general population, or other (studies using family members or case acquaintances as controls, or studies that used a combination of different sources)], geographical area (North America, Europe, or other), and whether adjustments were made for potential confounders. The *I*^2^ measure was used to quantify the heterogeneity between studies; *I*^2^ can be interpreted as a measure of the percentage of the total variation that cannot be explained by chance (Higgins et al. 2003). *p*-Values for heterogeneity are based on the *Q*-statistic. Small study effects were tested with funnel plots and Egger’s test (Egger et al. 1997). All analyses were performed with Stata (version 10; StataCorp, College Station, TX, USA) with the metan, metareg, metafunnel, and metabias commands. All statistical tests were two sided, and a *p*-value of < 0.05 was considered statistically significant.

## Results

The search in Embase and Medline yielded 883 publications, of which 52 publications met the inclusion criteria. We excluded 3 publications (Fong et al. 2005; Menegon et al. 1998; Smargiassi et al. 1998) where the study population had been included in subsequent publications (De Palma et al. 1998; Fong et al. 2007; McCann et al. 1998). Lastly, one study (Taylor et al. 1999) was excluded because the reported data showed risk per year of pesticide exposure, which was not comparable with reported risk ratios of other studies. Among the remaining 48 publications, there were two studies for which the relevant results were reported in two separate publications each (Firestone et al. 2005, 2010; Semchuk et al. 1992, 1993). Thus, results of a total of 46 studies were used in the meta-analysis.

An overview of the study characteristics of the included studies can be found in Table 1. There were 39 case–control studies, 4 cohort studies, and 3 cross-sectional studies; 40 publications reported on pesticides, 15 on herbicides, 15 on insecticides, and 9 on fungicides. Three studies included all parkinsonism (Duzcan et al. 2003; Engel et al. 2001a; Tanner et al. 2009); the rest studied idiopathic PD. Four studies showed only results in men (Engel et al. 2001a; Fall et al. 1999; Petersen et al. 2008; Petrovitch et al. 2002). One study included only cases with a disease diagnosis before 51 years of age (Butterfield et al. 1993), which is much lower than the average age of disease onset in all other studies (generally ~ 60 years of age). Information about participation rates was provided for only 13 of the 39 case–control studies. Studies that reported participation rates had rates between 69% and 100% for cases and between 41% and 100% for controls.

**Table 1:** Overview of the studies included in the meta-analyses.

Study	Study design	Location	Cases	Controls	Exposure assessment	Adjustments	Remarks
Ho et al. 1989		CC_o_		Hong Kong		35 PD patients Age range, 65–87 years		105 age/sex matched		SR-E/N Occ/Non-Occ P		—		—
Koller et al. 1990		CC_h_		USA		150 PD patients Age range, 39–87 years Mean age, 66 years		150 age/sex matched		SR-E/N Occ only P		—		OR calculated from reported numbers
Golbe et al. 1990		CC_o_		USA		106 PD patients No age information		106 spouses		SR-R Occ/Non-Occ P		—		OR calculated from reported numbers
Zayed et al. 1990		CC_p_		Canada		42 PD patients No age information		84 age/sex matched		SR-R Occ/Non-Occ P		Age, sex		—
Wong et al. 1991		CC_h_		USA		38 PD patients Mean age, 70 years		38 age/sex matched		SR-E/N Occ/Non-Occ P		—		OR calculated from reported numbers
Stern et al. 1991		CC_o_		USA		80 PD patients, diagnosed after 60 years of age No age information		80 age/sex/race/ participating center matched		SR-E/N Non-Occ only H, I		—		—
Jiménez-Jiménez et al. 1992		CC_h_		Spain		128 PD patients Mean age, 66.8 years		256 age/sex matched		SR-R Occ/Non-Occ P		—		OR calculated from reported numbers
Semchuk et al. 1992, 1993		CC_p_		Canada		130 PD patients Age range, 36–97 years Mean age, 68.5 years Participation, 88%		260 age/sex matched Participation, 76%		SR-E/N Occ only P, H, I, F		—		Herbicides OR adjusted for PD family history and head trauma
Hubble et al. 1993		CC_o_		USA		63 PD patients Mean age: urban patients, 69.3 years; rural patients, 69.0 years		75 with similar mean age		SR-R Occ/Non-Occ P		Age < 65 years; male; lifestyle; ethnicity; family history; fresh produce consumption; history of head trauma, depression or CNS infection		—
Butterfield et al. 1993		CC_o_		USA		63 PD patients, diagnosed before 51 years of age Age range, 35–72 years Mean age, 49 years Participation, 69%		68 age/sex/diagnosis year frequency matched Participation, 41%		SR-R Occ/Non-Occ H, I, F		Age, sex, race, age at diagnosis, education, family history		95% CIs calculated from ORs and *p*-values F-OR is not adjusted
Morano et al. 1994		CC_h_		Spain		74 PD patients Mean age, 68.4 years		148 age/sex matched		SR-R Occ/Non-Occ P		—		OR calculated from reported numbers
Hertzman et al. 1994		CC_p_		Canada		142 PD patients Mean age, 70.4 years		124 controls 45–80 years of age Participation, 61%		SR-E/N Occ only P, H, I, F		—		Reported results were pooled for men and women A second control group consisting of hospital controls was not used in this meta-analysis
*continued next page*														
Table 1. continued														
Study		Study design		Location		Cases		Controls		Exposure assessment		Adjustments		Remarks
Chaturvedi et al. 1995		CS		Canada		87 PD patients No age information		2,070 controls from cross-sectional study among elderly		SR-R Non-Occ only P		—		—
Seidler et al. 1996		CC_p_		Germany		379 PD patients < 66 years of age Mean age, 56.2 years Participation, 71%		379 age/sex matched		SR-E/N Occ/Non-Occ H, I		Smoking, education		The reported results for exposure categories were pooled A second control group consisting of neighborhood was not used in this meta-analysis
Liou et al. 1997		CC_h_		Taiwan		120 PD patients Age range, 37–91 years Mean age, 63.1 years		240 age/sex matched		SR-R Occ/Non-Occ P		—		—
De Palma et al. 1998		CC_h_		Italy		100 PD patients Mean age, 66.6 years		200 controls, similar in age and sex		JT Occ/Non-Occ P		—		Substantial leisure activities were also classified for exposure
Chan et al. 1998		CC_h_		Hong Kong		215 PD patients Age < 60 years, 13.5% Age 60–69 years, 33.5% Age 70–79 years, 33.5% Age ≥ 80 years, 19.5%		313 age/sex/hospital matched		SR-E/N Occ only P		Smoking, family history, rural living, well-water drinking, farming, consumption of tea, fruit vegetables and vitamins/liver oil supplements		Substantial difference between OR from unadjusted and adjusted analysis. Unadjusted OR = 1.80 (95% CI: 0.90, 3.58)
McCann et al. 1998		CC_o_		Australia		224 PD patients Mean age, 70.3 years		310 age/sex/ ethnicity/residential area/site of collection matched		SR-R Occ only P		—		—
Gorell et al. 1998		CC_p_		USA		144 PD patients 50 years or older Age 50–59 years, 9.0% Age 60–69 years, 30.6% Age 70–79 years, 46.5% Age ≥ 80 years, 13.9% Participation, 81%		464 ages/sex/race frequency matched Participation, 65%		SR-E/N Occ only, and Non-Occ only H, I, F		Age, sex, race, smoking		—
Werneck and Alvarenga 1999		CC_h_		Brasil		92 PD patients Age range, 55–78 years Mean age, 70.6 years		110 age/sex matched		SR-R Occ/Non-Occ P		—		—
Fall et al. 1999		CC_p_		Sweden		113 PD patients Age range, 40–75 years Mean age, 63.9 years Participation, 90%		263 from same age category Participation, 82%		SR-E/N Occ only P, I		Smoking, alcohol, coffee, and fried/ broiled meat consumption, carpenters, cabinetmakers		Only results for men are shown I-OR is not adjusted
Kuopio et al. 1999		CC_p_		Finland		123 PD patients Mean age, 69.3 years		246 age/sex/ municipality matched Participation, 68%		SR-E/N Occ only H		—		The reported results for “pesticides” do not contain herbicides and are not included in this review
Preux et al. 2000		CC_h_		France		140 PD patients Mean age, 71.1 years		280 age/sex matched		SR-E/N Occ/Non-Occ P		—		OR calculated from reported numbers
Herishanu et al. 2001		CC_h_		Israel		93 PD patients No age information		93 age/sex matched		SR-E/N Occ/Non-Occ P		Smoking, birth country, peptic disease, work in construction or in mechanical factory		—
Engel et al. 2001a		CS		USA		65 parkinsonism patients No age information		310 of original 1,300 men who previously participated in a cohort study		SR-E/N Occ only P, H, I, F		Age, smoking		The study was among men only
Behari et al. 2001		CC_h_		India		377 PD patients Age range, 24–86 years Mean age, 56.8 years Participation, 100%		377 age matched Participation, 100%		SR-E/N Occ/Non-Occ H, I		—		ORs calculated from reported numbers
Zorzon et al. 2002		CC_h_		Italy		136 PD patients Mean age, 70.0 years		272 age/sex matched		SR-E/N Occ/Non-Occ P		Smoking		
Petrovitch et al. 2002		Co		Hawaii		99 PD patients after 30 years of follow-up Median age at diagnosis, 73.7 years Range, 54–89 years		Baseline, 7,986 Japanese men in Hawaii		SR-R Occ/Non-Occ P		—		RR calculated from reported incidence numbers
Duzcan et al. 2003		CC_p_		Turkey		36 parkinsonism patients, > 50 years of age Age 50–59 years, 11.1% Age 60–69 years, 30.6% Age 70–79 years, 47.2% Age ≥ 80 years, 11.1%		108 age/sex matched		SR-R Occ/Non-Occ P		—		—
*continued next page*														
Table 1. continued														
Study		Study design		Location		Cases		Controls		Exposure assessment		Adjustments		Remarks
Baldereschi et al. 2003		CS		Italy		113 PD patients Mean age , 78.1 years		Study among 4,496 randomly selected elderly		SR-E/N Occ only P		Age, sex, education, smoking		Having a pesticide-use license was used as a proxy for pesticide use
Baldi et al. 2003a		CC_p_		France		84 PD patients, > 69 years of age Mean age, 75.6 years		252 age/sex matched		JT Occ only P		Age, sex, smoking, education		—
Baldi et al. 2003b		Co		France		24 PD patients after 5-year follow-up No age information		Baseline, 1,507 persons who were ≥ 65 years of age in specific area		JT Occ only P		Smoking, education		Reported results for men and women were pooled
Nuti et al. 2004		CC_p_		Italy		190 PD patients Mean age, 63.9 years		190 age/sex/ sociocultural factors matched		SR-E/N Occ/Non-Occ P		—		OR calculated from reported numbers
Frigerio et al. 2006		CC_p_		USA		149 PD patients Age range, 41–97 years Mean age, 70.0 years Participation, 76%		129 age/sex matched Participation, 66%		SR-E/N Occ/Non-Occ P, H, I		Age, sex		Also results occupational only (farming)
Ascherio et al. 2006		Co		USA		413 PD patients after 9-year follow-up Mean onset age, 70 years		Baseline: 184,190 persons		SR-R Occ/Non-Occ P		Age, sex, smoking, coffee, NSAIDs, education, physical activity		—
Kamel et al. 2007		Co		USA		78 PD patients after 5-year follow-up Age ≤ 50 years, 9% Age 51–60 years, 40% Age 61–70 years, 41% Age > 70 years, 10%		Baseline: 84,738 persons (applicants for pesticide use certification and their spouses)		SR-E/N Occ/Non-Occ P		Age, state, applicator or spouse		—
Dick et al. 2007		CC_o_		Scotland, Sweden, Romania, Italy, Malta		767 PD patients Mean age, 69.8 years Participation, 77%		1,989 age/sex/country frequency matched Participation, 59%		SR-E/N (+ JT) Occ/Non-Occ P		Age, sex, country, smoking, family history, ever knocked unconscious		—
Fong et al. 2007		CC_h_		Taiwan		153 PD patients Mean age, 71.7 years		155 age/sex/ birthplace matched		SR-R Occ only P		Age, sex, smoking		—
Brighina et al. 2008		CC_o_		USA		833 PD patients, Age range, 32–91 years Median age, 67.7 years		361 age/sex/region matched and 472 siblings		SR-R Occ/Non-Occ P, H, I, F		Age, sex		—
Hancock et al. 2008		CC_o_		USA		319 PD patients Age range, 29–94 years Mean age, 65.6 years		296 relatives and spouses		SR-E/N Occ/Non-Occ P, H, I		Age, sex, smoking, caffeine consumption		—
Petersen et al. 2008		CC_p_		Faroe islands		79 PD patients Mean age, 74.4 years		154 age/sex matched		SR-E/N Occ only P		Smoking		Only OR in men is shown because no exposed women in study
Elbaz et al. 2009		CC_p_		France		224 PD patients < 76 years of age Median age, 69.0 years Participation, 83%		557 age/sex/region matched Participation, 75%		SR-E/N Occ only, and Non-Occ only P, H, I, F		Smoking, Mini Mental State Examination score		Reported I-OR, H-OR, and F-OR for men and women were pooled The OR for Non-Occ only is unadjusted
Tanner et al. 2009		CC_o_		USA		519 parkinsonism patients Age range, 30–88 years Median age, 65 years		511 age/sex/ location frequency matched		SR-E/N Occ only P		Age, sex, ethnicity, smoking, alcohol, caffeine, head injury		—
Vlajinac et al. 2010		CC_h_		Serbia		110 PD patients Mean age, 60.8 years Participation, 100%		220 age/sex/urban or rural living matched Participation, 100%		SR-E/N Occ/Non-Occ P, H, I, F		I-OR is adjusted for gardening, rural living, well and spring water drinking, dyes or naphtha exposure, service-sector work		OR, H-OR, and F-OR calculated from reported numbers
Firestone et al. 2005, 2010		CC_h_		USA		404 PD patients Age range, 29–88 years Median age, 69 years Participation, 70%		526 age/sex frequency matched Participation, 60%		SR-E/N Occ only, and Non-Occ only P, H, I, F		Age, ethnicity, smoking		Reported results for all pesticides were pooled for men and women Only results for men are shown for the subgroups for Occ only
Manthripragada et al. 2010		CC_p_		USA		351 PD patients Age ≤ 60 years, 22% Age > 60 years, 78%		363 controls from same region		SR-E/N (+ JT) Occ only P		Age, sex, ethnicity, smoking, education, county		—
Abbreviations: CC_h_, case–control study with hospital controls; CC_o_, case–control study with controls from other sources or a combination of sources; CC_p_, case–control study with population controls; CNS, central nervous system; Co, cohort study; CS, cross-sectional study; F, fungicides; H, herbicides; I, insecticides; JT, job titles; Non-Occ only, only nonoccupational exposure included in the exposed group; NSAIDs, nonsteroidal anti-inflammatory drugs; Occ only, only occupational exposure included in the exposed group; Occ/Non-Occ, nonoccupational exposure included in the exposed group; P, pesticides; SR-E/N, self-report ever/never; SR-R, self-report regular.														

**Figure 1:**
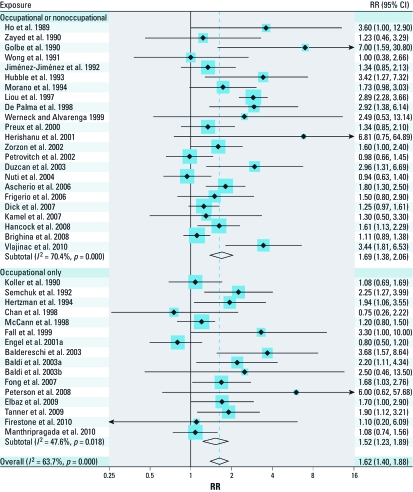
Forest plot for study-specific RRs and sRRs (95% CIs) of PD associated with the use of pesticides. The studies are ordered by publication year and stratified by studies that did or did not include nonoccupational exposure in the exposed group. Studies were pooled with the random effects method. The size of the squares reflects the statistical weight of the study in the meta-analyses.

Figure 1 shows PD relative risk estimates for any pesticide exposure based on studies of occupational and/or nonoccupational exposures, and studies of occupational exposures only. The summary risk ratios (sRRs) between these two groups were very similar, with sRRs of 1.69 (95% CI: 1.38, 2.06) and 1.52 (95% CI: 1.23, 1.89), respectively, and an overall sRR for all studies combined of 1.62 (95% CI: 1.40, 1.88). The *I*^2^ for all studies combined was 63.7%. Only three studies estimated effects of nonoccupational exposure only (Chaturvedi et al. 1995; Elbaz et al. 2009; Firestone et al. 2005), with an sRR of 1.18 (95% CI: 0.86, 1.63).

**Figure 2:**
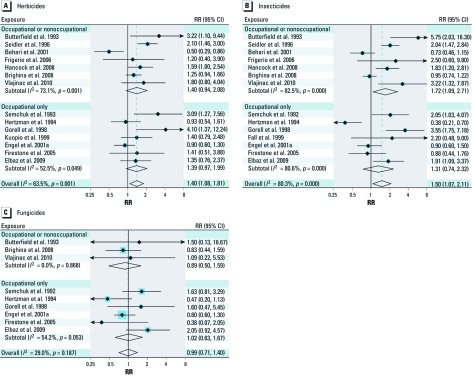
Forest plots for study-specific RRs and sRRs (95% CIs) of PD associated with the use of herbicides (*A*), insecticides (*B*), and fungicides (*C*). The studies are ordered by publication year and stratified by studies that did or did not include nonoccupational exposure in the exposed group. Studies were pooled with the random effects method. The size of the squares reflects the statistical weight of the study in the meta-analyses.

Meta-analyses by herbicide, insecticide, and fungicide exposure are shown in Figure 2. In line with the results for any pesticide exposure, we did not observe noticeable differences between studies of occupational exposures only and studies of nonocccupational and occupational exposures combined. The sRR for exposure to fungicides did not indicate an association with PD (overall sRR = 0.99; 95% CI: 0.71, 1.40; Figure 2C), in contrast with positive sRRs for exposure to herbicides (overall sRR = 1.40; 95% CI: 1.08, 1.81; Figure 2A) and insecticides (overall sRR = 1.50; 95% CI: 1.07, 2.11; Figure 2B).

**Figure 3:**
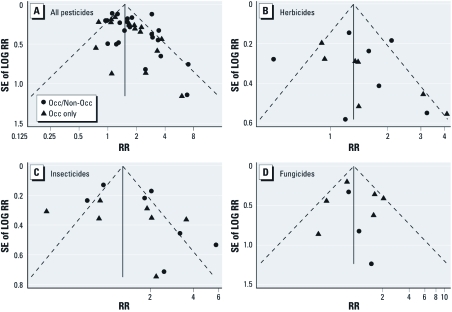
Funnel plots of studies included in the meta-analysis for the risk of PD associated with the use of pesticides (*A*), herbicides (*B*), insecticides (*C*), and fungicides (*D*). Circles represent studies that included nonoccupational exposure in the exposed group, and triangles represent studies that were based on occupational exposure only. Egger’s test *p*-values were 0.057, 0.338, 0.208, and 0.680 for pesticide, herbicide, insecticide, and fungicide effect estimates, respectively.

Funnel plots of effect estimates for exposure to pesticides and pesticide subcategories were suggestive of small study effects, with a tendency for smaller studies to report higher relative risks compared with larger studies (Figure 3), with Egger’s test *p*-values of 0.057, 0.338, 0.208, and 0.680 for pesticide, herbicide, insecticide, and fungicide effect estimates, respectively.

**Figure 4:**
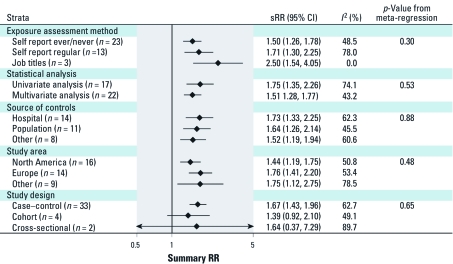
sRRs (95% CIs) for strata of exposure assessment method, statistical analysis, source of controls, study area, and study design. The *p*-value from meta-regression represents the *p*-value of the *F*-test in case of more than two categories, whereas it represents the *p*-value for the *t*-test in the case of the two statistical analysis strata.

Figure 4 presents subgroup sRR estimates for those factors *a priori* hypothesized to be related to the observed heterogeneity in study results. The only study characteristic that was suggestive of contributing to heterogeneity was the exposure assessment method, with the lowest summary estimates observed for self-reported exposures (*n* = 36) and highest sRR for studies with exposures estimated based on reported job titles (*n* = 3). However, these differences were not statistically significant (*p* = 0.30). There was no evidence for a difference in summary estimates by adjustment of results for potential confounders, type of control population source, geographical area, or by study design. We also investigated whether adjustment for smoking had an effect on the summary risk estimate. Almost identical results were found for studies that did or did not correct for smoking (data not shown). Similar analyses for the subcategories herbicides and insecticides rendered similar results as for all pesticides (data not shown).

## Discussion

Our systematic review indicated that PD is related to pesticide exposure with an sRR of 1.62 (95% CI: 1.40, 1.88). However, there was substantial heterogeneity among individual study estimates (*I*^2^ = 63.7%). Summary estimates also indicated positive associations of PD with herbicides and insecticides, but not with fungicides. We systematically investigated several factors that could explain heterogeneity in study results, but none appeared to be related to the observed heterogeneity, with the possible exception of the method of exposure assessment. Studies that based their exposure assessment on job titles reported somewhat higher risk estimates than studies that used self-reported exposures, but the difference did not reach statistical significance, in part because of low numbers of studies relying on job title and expert judgment.

Including persons who were nonoccupationally exposed to pesticides together with those occupationally exposed resulted in a very similar sRR. Given that occupational pesticide applications are in general more frequent and on larger areas than are nonoccupational exposures, one would have anticipated higher RRs for studies focusing only on occupational exposures. On the other hand, use of protective equipment during nonoccupational applications may be less. The fact that summary results were similar for both types of studies could indicate that nonoccupational and occupational pesticide exposures carry similar risks, or that most of the exposures in the combined studies were occupational. In the three studies that exclusively reported on nonoccupational pesticide exposures, only a small increase in relative risk was observed (sRR = 1.18; 95% CI: 0.86, 1.63), suggesting that risks associated with nonoccupational pesticide exposures are lower than from occupational exposures. Nevertheless, nonoccupational pesticide exposure cannot be ruled out as a risk factor for PD based on these analyses.

Studies used different methods for exposure assessment and assignment. Most studies (36 of 39) were based on self-reported exposure to pesticides, defined as ever versus never use or as regular versus nonregular use. No difference in sRR was seen between these two definitions of self-reported exposure, although it could have been expected that using a more stringent definition of exposure would have resulted in stronger associations. Studies that used reported job titles and expert judgment, and/or that used a job-exposure matrix to estimate exposures, resulted in a higher sRR compared with studies using self-reported pesticide exposures. This difference cannot be explained by recall bias, because in that case, higher risk ratios would have been expected for studies relying on self-reported exposures. A more likely explanation is that subjects are not able to reliably report exposures to pesticides, resulting in nondifferential exposure misclassification and bias toward the null (Daniels et al. 2001; Engel et al. 2001b). The fact that some heterogeneity is observed in study results by exposure assessment method indicates that this may be an important factor that should be taken into consideration when designing or interpreting studies.

A broad range of different pesticides exist with different chemical compositions and working mechanisms. In line with the conclusions of Brown et al. (2006), we found that both herbicides and insecticides, but not fungicides, were associated with PD. However, it is difficult to disentangle the effect of herbicides and insecticides given that the use of these two pesticide groups is often highly correlated. This is illustrated by the fact that we observed a correlation coefficient of 0.79 between the study-specific RRs of herbicides and insecticides. Few studies have focused on specific pesticides precluding any meaningful meta-analyses (Brighina et al. 2008; Elbaz et al. 2009; Engel et al. 2001a; Firestone et al. 2010; Hancock et al. 2008; Hertzman et al. 1994; Kamel et al. 2007; Liou et al. 1997; Seidler et al. 1996; Semchuk et al. 1992; Tanner et al. 2009; Vlajinac et al. 2010). However, it is interesting to note that the subgroup of organochlorines was significantly associated with PD in three studies (Elbaz et al. 2009; Hancock et al. 2008; Seidler et al. 1996). This is also in line with studies on biomarkers in serum (Richardson et al. 2009; Weisskopf et al. 2010) and in the brains of deceased patients (Corrigan et al. 2000; Fleming et al. 1994). Organochlorines are mainly insecticides, including DDT (dichlorodiphenyltrichloroethane), dieldrin, and heptachlor.

Funnel plots gave some indication for a small-study effect, such that larger effect estimates appeared to be associated with smaller studies, which suggests that the sRR might be slightly overestimated. In addition, the studies included were generally small, resulting in imprecise effect estimates that could have contributed to the substantial heterogeneity in study results. Meta-regression analyses provided no evidence for a difference in sRRs based on study design, geographical area, adjustment for potential confounders, or type of control population. As such, factors explaining the heterogeneity observed remain largely elusive. We were not able to investigate the effect of differences in criteria used for the diagnosis of PD because there was substantial variation in the exact inclusion criteria among the studies that reported on the criteria used. However, in most of the studies, the diagnosis was made by a physician and included the presence of two or three of the cardinal symptoms of PD, often with some additional inclusion and exclusion criteria. Variations in participation rates could also contribute to study heterogeneity. The ability to investigate this factor was limited because only 13 of the case–control studies reported participation rates. The same is true for differences across sex. Only 8 studies showed separate results for men and women, but the results were not conclusive: RRs were higher for men than for women in 3 studies (Baldi et al. 2003b; Frigerio et al. 2006; Hertzman et al. 1994), higher for women than men in 3 other studies (Chan et al. 1998; Elbaz et al. 2009; Firestone et al. 2010), and comparable between men and women in the remaining 2 studies (Ascherio et al. 2006; Brighina et al. 2008). Heterogeneity in the results could also arise from both quantitative and qualitative differences in types of agriculture in the study areas. Although we compared large regions (i.e., North America, Europe, and other), this analysis would not have captured regional differences in the types of agriculture and pesticides used. Analyses by time periods might provide some clues because pesticide use has changed over the decades, but data were insufficient to perform a meaningful analysis of changes over time.

## Conclusion

Our overall summary risk estimates strongly suggest that exposure to pesticides, and to herbicides and/or insecticides in particular, increases the risk of developing PD. Heterogeneity among study-specific RRs could not easily be explained by methodological differences, except for a suggestive effect of exposure assessment characteristics. Future studies should therefore focus on using more objective semiquantitative methods for exposure assessment such as job- or crop-exposure matrices, rather than relying solely on self-report. Although classes of pesticides have been linked to PD, it remains important to identify the specific chemicals responsible for this association. Therefore, in new, preferably prospective studies, attention should be given to collecting detailed information on specific pesticide use.
